# Cost-effectiveness of lurbinectedin with atezolizumab for extensive-stage small-cell lung cancer in the United States: impact of drug wastage reduction on economic outcomes

**DOI:** 10.1186/s12913-026-14653-5

**Published:** 2026-05-04

**Authors:** Jiaming Zhu, Zhengxiong Li, Danlei Song, Wen Liu

**Affiliations:** 1 Fourth Clinical Medical College of Zhejiang Chinese Medical University, Affiliated Hangzhou First People’s Hospital, Hangzhou, 310053 P.R. China; 2 School of Medical Informatics and Engineering, Xuzhou Medical University, Xuzhou, 221004 P. R. China; 3 School of Humanities and Management, Zhejiang Chinese Medical University, Hangzhou, 310053 P.R. China

**Keywords:** Cost-effectiveness, Extensive-stage small-cell lung cancer, Lurbinectedin, Atezolizumab, Drug wastage and mitigation

## Abstract

**Background:**

The IMforte trial had supported lurbinectedin as a recommended first-line maintenance therapy for patients with extensive-stage small-cell lung cancer (ES-SCLC), while its cost-effectiveness remains uncertain for patients and clinical decision-makers. This study aims to evaluate the cost-effectiveness of adding lurbinectedin to atezolizumab as maintenance therapy for ES-SCLC and to assess the impact of drug wastage and mitigation strategies from the perspective of the US healthcare system.

**Methods:**

A partitioned survival model was developed to compare the cost-effectiveness of lurbinectedin plus atezolizumab versus atezolizumab alone. Key outcomes included total costs, effectiveness measured in quality-adjusted life years (QALYs), and the incremental cost-effectiveness ratio (ICER). Drug wastage was incorporated in the base-case analysis, and scenario analyses evaluated mitigation strategies through vial sharing and dose rounding. Model uncertainty was assessed using one-way sensitivity analysis and probabilistic sensitivity analysis.

**Results:**

Over a 10-year time horizon, the lurbinectedin plus atezolizumab group accrued 0.3 more QALYs than the atezolizumab group. Under conditions of drug wastage, the addition of lurbinectedin resulted in an incremental cost of $230,000. When drug wastage was either excluded or mitigated through strategies such as vial sharing and dose rounding, the incremental cost was reduced to $180,000. The corresponding ICERs were $770,000 and $610,000/QALY, respectively. To achieve a greater than 50% probability of cost-effectiveness, an 85% price reduction in both high-cost drugs would be required. Sensitivity analyses indicated that the results were robust to variations in model parameters.

**Conclusions:**

At current price, lurbinectedin plus atezolizumab is not cost-effective as ES-SCLC maintenance therapy. Mitigating drug wastage and implementing substantial price reductions are essential to improve its economic value.

**Supplementary Information:**

The online version contains supplementary material available at 10.1186/s12913-026-14653-5.

## Introduction

Approximately 30,000 to 35,000 new cases of small cell lung cancer (SCLC) are diagnosed annually in the United States (US), accounting for roughly 13% of all lung cancer diagnoses [1]. SCLC is characterized by rapid proliferation, early metastasis, and widespread disease at initial diagnosis, contributing to its poor prognosis. The 5-year survival rate remains below 7% [2]. The aggressive and disseminated nature of the disease presents significant therapeutic challenges, particularly in extensive-stage SCLC (ES-SCLC), where surgical resection is rarely feasible. Chemotherapy remains the cornerstone of treatment, with the combination of a platinum-based chemotherapy and etoposide (EP) being the most commonly used regimen. Although initial responses to chemotherapy are often favorable, most patients ultimately develop resistance and experience disease recurrence, rendering the benefits of treatment short-lived. In recent years, immunotherapy has brought meaningful advances in SCLC management. The addition of atezolizumab or durvalumab to EP has demonstrated significant improvements in progression-free survival (PFS) and overall survival (OS), and is now recommended by the National Comprehensive Cancer Network (NCCN) guidelines as a first-line treatment option for ES-SCLC [3].

Unlike traditional topoisomerase inhibitors such as topotecan and etoposide, lurbinectedin is a ribosomal transcription inhibitor that binds to DNA and blocks the activity of RNA polymerase II, thereby disrupting the transcriptional process. In a phase II clinical trial, lurbinectedin demonstrated promising efficacy in patients with chemotherapy-resistant ES-SCLC [4]. The phase III ATLANTIS clinical trial evaluated the effectiveness of lurbinectedin in combination with topotecan in patients with ES-SCLC [5]. Although the study did not meet its primary endpoint with statistical significance, the combination therapy showed potential benefits in specific patient subgroups compared to traditional chemotherapy. Based on these findings, lurbinectedin was approved by the US Food and Drug Administration (FDA) in 2020 as a second-line treatment for ES-SCLC, offering an option for patients with resistance to topoisomerase inhibitors. Notably, lurbinectedin also exhibits immunomodulatory properties by inhibiting tumor-associated macrophages and activating the stimulator of interferon genes - interferon signaling pathway, thereby restoring T-cell function and enhancing the efficacy of immunotherapy [6–8]. The phase III IMforte trial confirmed the clinical benefit of lurbinectedin plus atezolizumab as a maintenance therapy for patients previously treated with atezolizumab plus EP. The combination regimen extended PFS by 3.3 months compared to monotherapy, without introducing new safety concerns, indicating a manageable safety profile [9].

Given the clinical benefits demonstrated in the IMforte trial and the current shortage of essential chemotherapeutic agents such as cisplatin and carboplatin in the United States [10], lurbinectedin may drive a paradigm shift in the maintenance treatment landscape for ES-SCLC, emerging as a potential first-line option. However, both lurbinectedin and atezolizumab are high-cost novel therapies, and the body surface area (BSA)-based dosing of chemotherapeutic agents often leads to substantial drug wastage, raising additional concerns regarding economic burden. Therefore, leveraging data from the IMforte trial, this study aims to evaluate the cost-effectiveness of the combination regimen versus monotherapy as a first-line maintenance strategy for ES-SCLC, from the perspective of the US healthcare system.

## Methods

Implementation of the Consolidated Health Economic Evaluation Reporting Standards (CHEERS) 2022 guidelines governed this investigation’s reporting quality. Item-by-item validation against the 28-criterion checklist is systematically presented in Table S1 in Supplement Materials.

### Patient population and therapy

Based on the global, multicenter IMforte clinical trial, this study simulated treatment outcomes in a hypothetical cohort of 65-year-old patients in US context. Patients were diagnosed with ES-SCLC confirmed by histology or cytology, and initially received a 4-cycle induction regimen of atezolizumab in combination with EP. Patients with prior exposure to immune checkpoint inhibitors or with central nervous system metastases were excluded from induction. Those who completed induction therapy without disease progression entered the maintenance phase and were randomized 1:1 to receive either lurbinectedin plus atezolizumab (Lu group) or atezolizumab alone (Ate group). The maintenance cycle length was set to 21 days, consistent with the drug administration schedule. Patients in the Lu group received intravenous lurbinectedin at 3.2 mg/m² once per cycle. Both groups received 1200 mg intravenous atezolizumab once per cycle until disease progression or the occurrence of unacceptable toxicity [9]. Upon disease progression, patients received subsequent anti-tumor therapy with agents such as carboplatin and etoposide. Those who did not receive subsequent anti-cancer therapy were considered to have received best supportive care (BSC). The proportion of patients receiving subsequent anti-cancer therapy, drug dosing schedules, incidence of adverse events, and follow-up protocols were derived from the IMforte trial and the NCCN Clinical Practice Guidelines (Version 1.2026) and were displayed in the Table S2-4 in Supplement Materials [3].

### Model overview

**Fig. 1:**
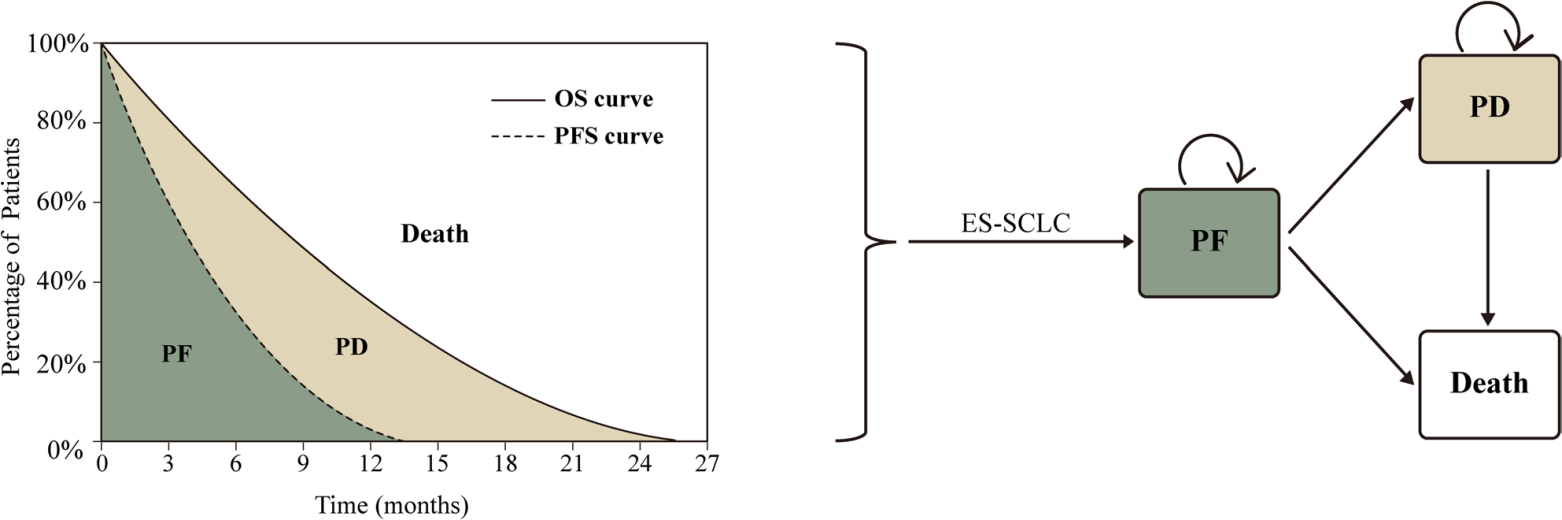
Partitioned survival model structure. Arrows represent transitions from one state to another. Abbreviations: PF: progression-free, PD: progressive disease

A partitioned survival model was developed using TreeAge Pro 2022 (TreeAge Software LLC) to assess the cost-effectiveness of the Lu strategy versus the Ate strategy in ES-SCLC patients receiving maintenance therapy. The model estimated both costs and outcomes associated with each treatment. All patients entered the model in the progression-free (PF) health state and remained in this state until disease progression or death, starting from the initiation of maintenance therapy (Fig. 1). The model was driven by PFS and OS Kaplan-Meier (KM) curves derived from the IMforte trial. The proportion of patients in each health state was estimated over time, and total costs and health outcomes were accumulated accordingly for economic comparison. Given the reported 10-year survival rate of 1.2% in ES-SCLC patients [11], the time horizon was set at 10 years, with a cycle length of 21 days. Model outcomes included total cost, total quality-adjusted life years (QALYs), and the incremental cost-effectiveness ratio (ICER). To evaluate economic acceptability, a WTP threshold of $100,000 to $150,000 per QALY was applied.

### Clinical data

Survival data were extracted from the KM curves reported in the IMforte trial using GetData Graph Digitizer (version 2.26), and individual patient data (IPD) were reconstructed in R (version 4.3.2) based on the algorithm proposed by Guyot et al. [12]. The restructuring results are available at Figure S1 in Supplement Materials. The proportional hazards assumption between treatment arms was evaluated based on reconstructed KM curves and was not supported. Therefore, independent parametric models were fitted for each treatment arm to account for non-proportional hazards. Seven standard parametric models including exponential, weibull, gamma, generalized gamma, Gompertz, log-logistic, and log-normal, and three flexible models including fractional polynomial (FP), restricted cubic spline (RCS), and Royston-Parmar (RP) spline models were fitted to the reconstructed IPD [13]. Model fit was evaluated using the Akaike Information Criterion (AIC) and visual inspection. The final model was selected based on both statistical goodness-of-fit and clinical plausibility. Due to the lack of long-term survival data beyond the follow-up period of the trial, the best-fitting model was used to extrapolate survival over the full 10-year time horizon of the economic model. The final survival functions for lurbinectedin group and atezolizumab group are presented in Table 1. Model fit statistics are provided in Table S5 and Figure S2-S3 in Supplement Materials.

**Table 1:** Model key parameters

Parameters	Base-case	Low	Upper	Distribution	Reference
**Clinical input**					
OS of lurbinectedin: RCS1	(Intercept)= -0.42; s(Time).1 = 0.58; s(Time).2 = 0.97				
OS of atezolizumab: Log-logistic	Shape = 1.82; Scale = 0.90				
PFS of lurbinectedin: RP-hazard-1	knot=3; gamma0 = 9.25; gamma1 = 4.68; gamma2 = 1.02; gamma3=-1.57; gamma4 = 0.94				
PFS of atezolizumab: RP-normal-1	knot=1; gamma0 = 4.42; gamma1 = 2.10; gamma2 = 0.09				
**Price of drug**					
Lurbinectedin per 4 mg	8,250.00	6,600.00	9,900.00	Gamma	RED BOOK [14]
Atezolizumab per 1200 mg	11,589.36	9,271.49	13,907.23	Gamma	RED BOOK [14]
Carboplatin per 50 mg	6.28	5.02	7.54	Gamma	RED BOOK [14]
Etoposide per 100 mg	10.30	8.24	12.36	Gamma	RED BOOK [14]
Topotecan per 4 mg	140.00	112.00	168.00	Gamma	RED BOOK [14]
Irinotecan per 100 mg	30.31	24.25	36.37	Gamma	RED BOOK [14]
Chemotherapy iv infusion 1 h	123.94	102.87	166.55	Gamma	CPTcodes 96413 [15]
**Cost of follow up of patients per unit**					
Functional MRI	258.07	91.87	508.44	Gamma	CPTcodes 70554 [15]
PET CT	111.90	104.52	164.79	Gamma	CPTcodes 78816 [15]
Blood-test	10.56	8.45	12.67	Gamma	CPTcodes 80053 [15]
Breathing capacity test	27.42	23.36	35.65	Gamma	CPTcodes 94010 [15]
Cost of best supportive care per cycle	1,551.00	1,240.80	1,861.20	Gamma	[16]
Cost of terminal care per patient	23,155.67	18,524.54	27,786.80	Gamma	[16]
**Cost of managing adverse events (grade 3-4) per event**					
Anaemia	9,125.12	7,300.10	10,950.14	Gamma	[17]
Decreased neutrophil count	14,637.49	11,709.99	17,564.99	Gamma	[16]
Decreased platelet count	14,046.89	11,237.51	16,856.27	Gamma	[16]
Neutropenia	15,377.85	12,302.28	18,453.42	Gamma	[17]
Thrombocytopenia	14,062.52	11,250.02	16,875.02	Gamma	[17]
Body surface (m^2^)	1.82	1.70	2.50	Gamma	[17, 26]
**Health state utilities**					
Progressive disease	0.70	0.56	0.84	Beta	[20]
Progression-free survival	0.75	0.60	0.90	Beta	[20]
**Disutility**					
Anaemia	0.07	0.06	0.08	Beta	[16]
Decreased neutrophil count	0.2	0.16	0.24	Beta	[21]
Decreased platelet count	0.05	0.04	0.06	Beta	[16]
Neutropenia	0.09	0.07	0.11	Beta	[16]
Thrombocytopenia	0.25	0.20	0.30	Beta	[17]
**Annual discount rate**	0.03	0.00	0.08	Fixed	[22]

Abbreviation: CT: computed tomography; MRI: magnetic resonance imaging

### Cost and utility

This study adopted the perspective of the US healthcare system, incorporating direct medical costs, including drug acquisition, follow-up examinations, and adverse events (AEs) management. Drug costs were based on wholesale acquisition costs from the RED BOOK [14]. Intravenous administration, follow-up examinations, and other healthcare services were costed using average 2024 rates from the Centers for Medicare & Medicaid Services (CMS) [15]. Costs for BSC, end-of-life care, and AE management were derived from previously published studies [16, 17]. Only grade 3-4 AEs were included in the cost analysis, calculated as the product of the reported per-event treatment cost and the incidence rate. All literature-derived costs were adjusted to 2024 US dollars (USD) using the Consumer Price Index for Medical Care [18] (Table 1). Given that lurbinectedin was supplied in single-dose vials and the product label mandates discarding any unused portion, drug wastage was explicitly modeled as a cost component. Individual doses were first calculated based on BSA, then rounded up to the nearest available vial size for procurement and billing purposes. In line with CMS single-dose vial billing policy, the total cost to the payer included both the administered dose and the discarded portion. The base-case analysis assumed no vial sharing or cross-patient pooling. Drug wastage was uniformly quantified using both the wastage rate and the monthly per-patient cost (in USD) and milligrams of wasted drug [19] (Table S2 in Supplement Materials).

Effectiveness was measured in QALYs. In each cycle, health utility was calculated and discounted accordingly. The total QALYs were obtained by summing the discounted utility over the entire time horizon. Utility values consisted of three components: utility during the PF state, utility after progression, and disutility from related AEs. Owing to the absence of utility data specific to ES-SCLC, published EQ-5D-5 L data from US patients with non-small cell lung cancer (NSCLC) were adopted as proxy values [20], consistent with previous economic evaluations in this setting [16]. The impact of this assumption on the ICER was tested in sensitivity analyses. Disutility values associated with grade 3-4 AEs were extracted from published literature [16, 17, 21]. For included AEs, expected disutilities per cycle were calculated as the product of the reported incidence rates in the IMforte trial and corresponding disutility weights. AE-related disutilities were applied as one-time decrements in the first treatment cycle. Both costs and QALYs were discounted at an annual rate of 3% [22].

### Sensitivity analysis

To assess parameter uncertainty, both one-way sensitivity analysis (OWSA) and probabilistic sensitivity analysis (PSA) were conducted. The OWSA evaluated the impact of individual model inputs on the ICER. For parameters lacking reported ranges, such as drug prices and AE management costs, a variation of ± 25% around the base-case estimates was applied. For follow-up and administration costs, the highest and lowest values from the 2024 cm database were used as upper and lower bounds, respectively [15]. Discount rate was varied between 0% and 8% [22]. A total of 10,000 Monte Carlo simulations were conducted in the PSA, with parameters jointly sampled from predefined distributions. Beta distributions were assigned to utility values, incidence of AEs and proportions of patients receiving subsequent anti-cancer treatments, while Gamma distributions were applied to cost-related parameters. For parameters without reported uncertainty, standard errors were assumed to be 10% of the point estimate, and distribution parameters were derived using method-of-moments estimation. All assumptions and distributions were consistent with best practices for decision-analytic modeling as recommended by the ISPOR [23].

### Scenario analyses

To explore the impact of drug pricing and drug wastage on cost-effectiveness outcomes, a series of scenario analyses were conducted. First, joint price reductions for lurbinectedin and atezolizumab were modeled by proportionally decreasing the acquisition cost of both agents to estimate ICER variations and define a value-based pricing range. Second, an ideal “zero-waste” scenario was simulated, assuming no cost from discarded drug in single-dose vials, to assess the resulting ICER. Based on this scenario, joint price reductions were re-applied to characterize the interaction between pricing and drug wastage.

Recognizing that drug wastage is difficult to eliminate in real-world settings, additional mitigation strategies were evaluated, including vial sharing (VS), dose rounding, and their combination. VS scenarios were modeled separately for high-volume cancer centers and outpatient chemotherapy clinics settings. For the cancer centers scenario, capacity was anchored to the chemotherapy scheduling volume of The University of Texas MD Anderson Cancer Center to estimate feasible sharing levels. For outpatient chemotherapy clinics, daily chemotherapy demand was estimated based on infusion room throughput reported by Slocum et al., in conjunction with ES-SCLC prevalence data [24]. To assess the cost-effectiveness implications of dose rounding, a Monte Carlo simulation was conducted to incorporate uncertainty in BSA. Dose rounding was assumed to be within ± 10% of the prescribed dose, based on recommendations from the Oncology Pharmacy Association [25]. BSA values were sampled 10,000 times from a Gamma distribution ranging from 1.70 to 2.50 m², consistent with the 5th to 95th percentile range reported for US male patients in the literature [26]. For each sample, drug dosage was calculated based on BSA, then adjusted according to the rounding rule and vial size to determine drug usage and cost. Scenario results for dose rounding were summarized as the median incremental cost and incremental QALY to reduce the influence of outliers.

Additionally, to assess the sensitivity of the model to the selected time horizon and survival modeling approach, scenario analyses were conducted using time horizons of 5, 15, and 20 years, as well as alternative parametric survival models for curve fitting and extrapolation.

## Result

### Base-case results

Over a 10-year time horizon, patients in the Lu group accrued 1.26 QALYs, representing a gain of 0.30 QALYs compared to 0.96 QALYs in the Ate group. However, the addition of lurbinectedin increased the total cost from $153,433.87 to $382,776.03, resulting in an incremental cost of $229,342.16. Consequently, the ICER for the combination therapy was $773,428.44/QALY, which exceeds the predefined WTP threshold. Therefore, at current prices, lurbinectedin in combination with atezolizumab is not considered cost-effective for maintenance therapy in ES-SCLC. Further analysis revealed that 88% of the total cost in the Lu group was incurred during the PF state, while only 35% of QALYs were accumulated in this state, indicating a relatively low conversion of expenditures into survival benefit during PF. To achieve cost-effectiveness at the $150,000/QALY threshold, the prices of lurbinectedin and atezolizumab required to be cost-effective were $1,212.75/ 4 mg and $1,703.63/ 1200 mg, respectively, compared to their current market prices of $8,250/ 4 mg and $11,589.36/ 1200 mg (Table 2).

**Table 2:** Summary of base-cases and scenario analysis results

Scenario	Cost ($)	Incremental Cost ($)	QALYs	Incremental QALYs	ICER ($/QALY)	The price reduction required for Lu to be cost-effective when WTP = $100,000/QALY	The price reduction required for Lu to be cost-effective when WTP = $150,000/QALY
Base-case:10-year horizon + drug wastage						92.2%	85.3%
Ate	153,433.87	NA	0.96	NA	NA	NA	NA
Lu + Ate	382,776.03	229,342.16	1.26	0.30	773,428.44	NA	NA
Scenario 1:10-year horizon +without drug wastage						89.5%	80.2%
Ate	153,125.98	NA	0.96	NA	NA	NA	NA
Lu + Ate	332,888.86	179,762.88	1.26	0.30	606,228.36	NA	NA
Scenario 2: 5-year horizon +drug wastage						94.0%	89.0%
Ate	140,212.65	NA	0.90	NA	NA	NA	NA
Lu + Ate	371,008.67	230,796.03	1.07	0.17	1,336,736.33	NA	NA
Scenario 3:15-year horizon +drug wastage						87.0%	78.0%
Ate	158,934.93	NA	0.99	NA	NA	NA	NA
Lu + Ate	390,089.28	231,154.36	1.40	0.42	554,559.65	NA	NA
Scenario 4:20-year horizon +drug wastage						83.0%	70.0%
Ate	161,734.65	NA	1.00	NA	NA	NA	NA
Lu + Ate	396,261.81	234,527.16	1.52	0.53	444,522.09	NA	NA
Scenario 5:20-year horizon +without drug wastage						77.0%	65.0%
Ate	161,425.52	NA	1.00	NA	NA	NA	NA
Lu + Ate	346,273.76	184,848.24	1.52	0.53	350,360.81	NA	NA
Scenario 6:10-year horizon +drug wastage +rounding dose						88.9%	80.0%
Ate	153,433.87	NA	0.96	NA	NA	NA	NA
Lu + Ate	349,245.38	195,811.51	1.26	0.30	660,350.41	NA	NA
Scenario 7:10-year horizon +Vial Sharing^†^						90.0%	82.1%
Ate	153,433.87	NA	0.96	NA	NA	NA	NA
Lu + Ate	334,789.72	181,355.85	1.26	0.30	611,600.47	NA	NA
Scenario 8:10-year horizon +Vial Sharing +Drug Day^‡^						90.4%	82.6%
Ate	153,433.87	NA	0.96	NA	NA	NA	NA
Lu + Ate	337,159.88	183,726.01	1.26	0.30	619,593.53	NA	NA

†: Large cancer centers, characterized by high chemotherapy demand and concentrated patient volumes, where the feasibility of vial sharing is relatively high

‡: Outpatient chemotherapy settings, with lower patient volumes and more dispersed demand, where the extent of vial sharing is limited

Abbreviation: Lu: lurbinectedin, Ate: atezolizumab, WTP: willingness-to-pay; QALY: quality-adjusted life year; ICER: incremental cost-effectiveness ratio

### Sensitivity analysis

**Fig. 2:**
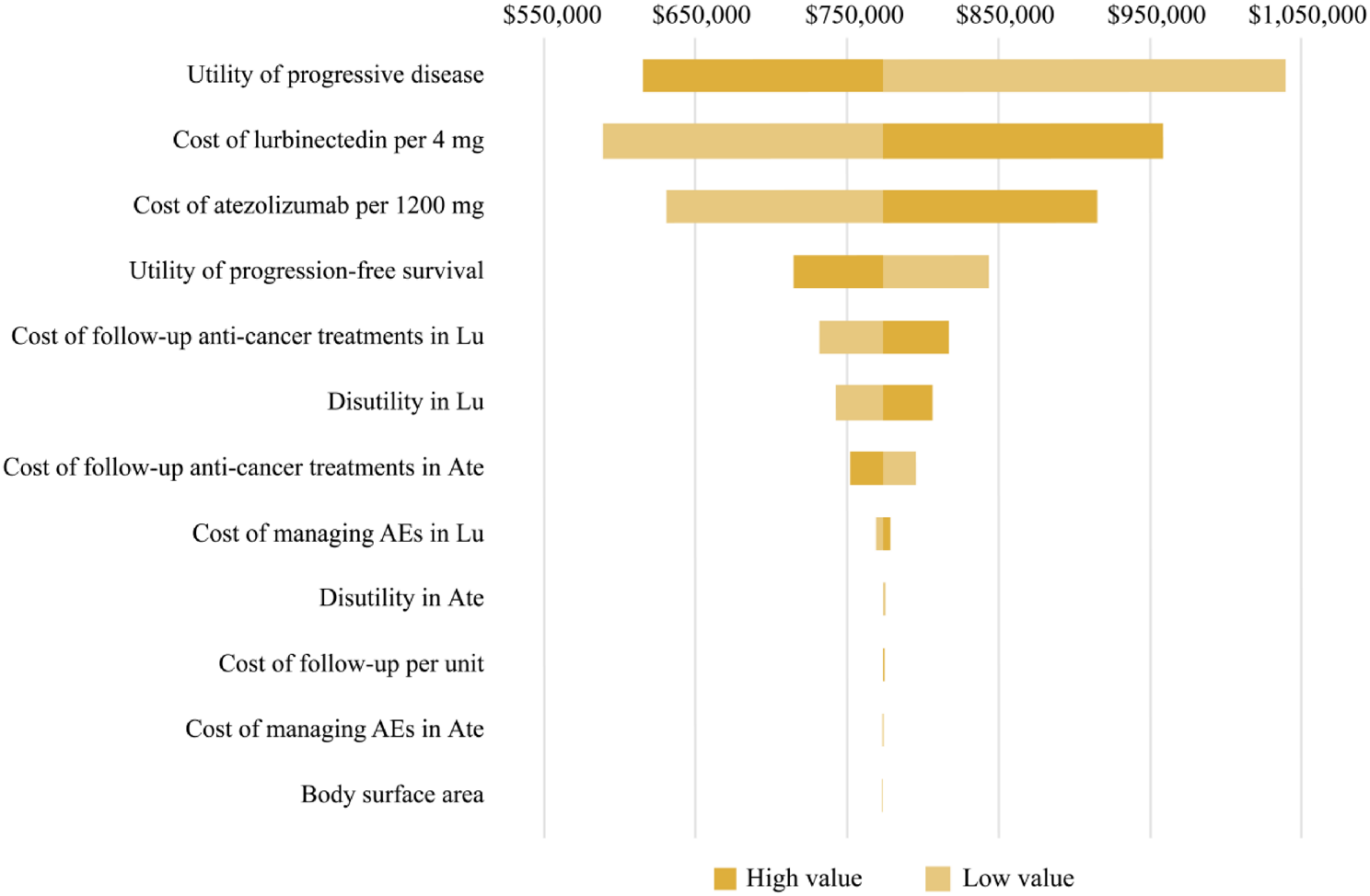
Tornado diagram of one-way sensitivity analysis. Abbreviations: Lu: lurbinectedin group; Ate: atezolizumab group; AEs: adverse events

OWSA indicated that the most influential parameters affecting the ICER were the utility value in the progressive disease (PD) state, the prices of lurbinectedin and atezolizumab, the utility value in the PF state, and the post-progression treatment cost in the Lu group. A 30% reduction in the price of lurbinectedin produced the largest decrease in the ICER, reducing it by 24%. However, this change was insufficient to alter the overall conclusion regarding cost-effectiveness (Fig. 2).

**Fig. 3:**
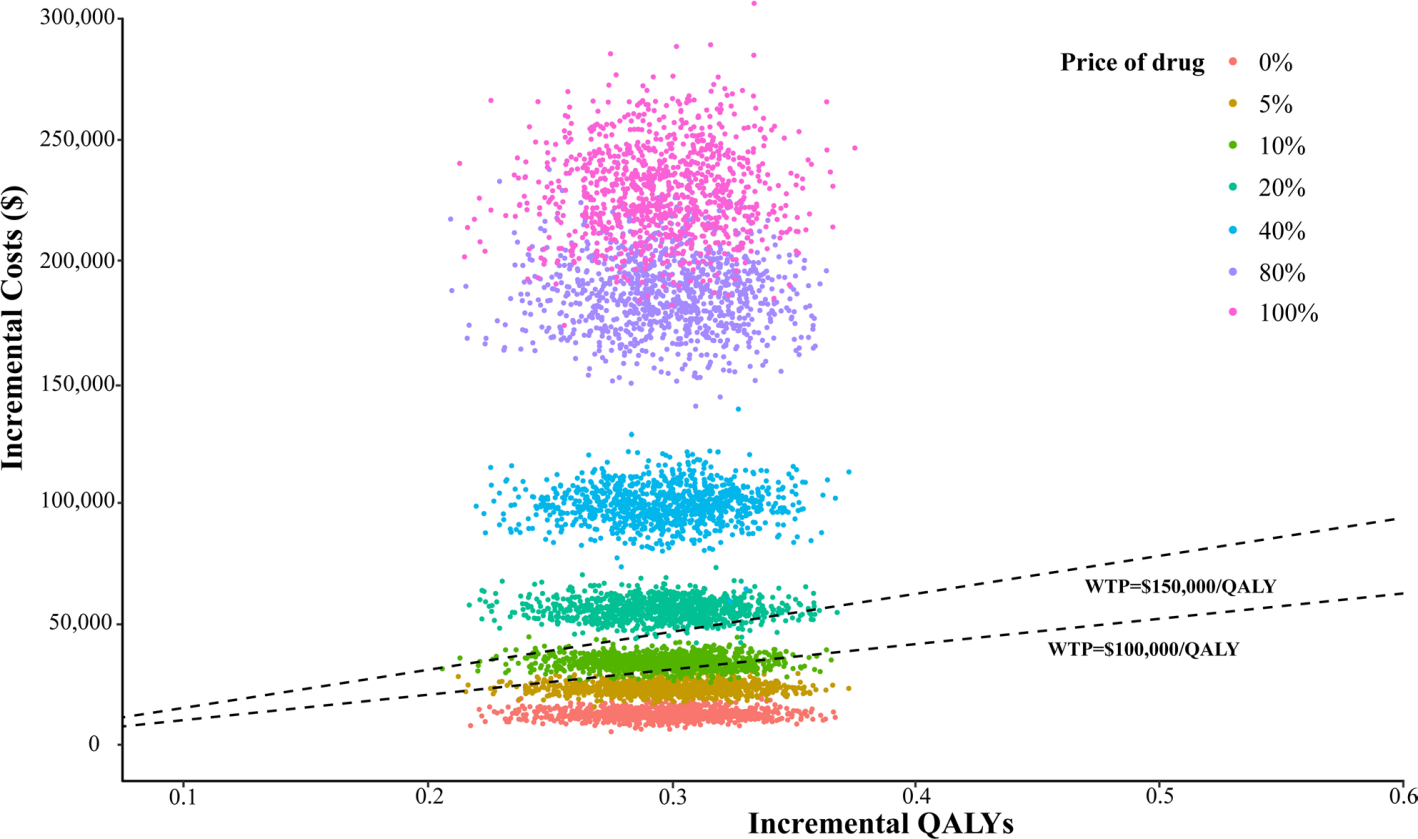
Cost-effectiveness scatterplot at multiple specific simulated prices. Abbreviations: WTP: willingness-to-pay; QALY: Quality-adjusted life year

**Fig. 4:**
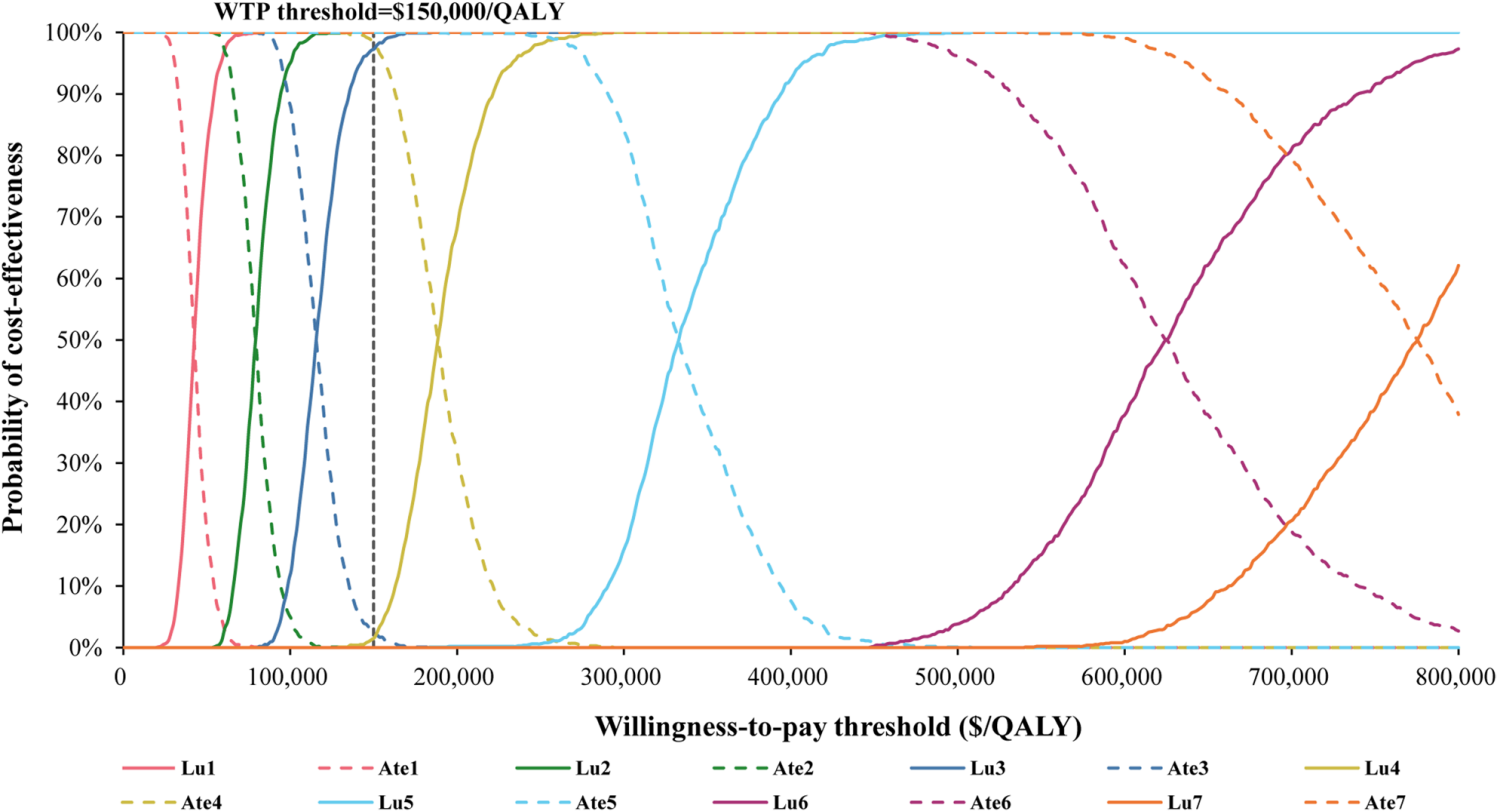
Cost-effectiveness acceptability curves at multiple specific simulated prices. Abbreviations: WTP: willingness-to-pay; QALY: Quality-adjusted life year; Lu: lurbinectedin; Ate: atezolizumab; Note: 0–7 refers to the price of lurbinectedin and atezolizumab change from 0%, 5%, 10%, 20%, 40%, 80% to 100%

PSA demonstrated that at current drug prices, the probability of the Lu strategy being cost-effective was 0% at a WTP threshold of $150,000 per QALY. Scatter plots indicated that when drug prices remained above 20% of the original level, the combination strategy was not cost-effective at the $150,000 threshold. When prices decreased to 10% of the original, most iterations fell between the $100,000 and $150,000 thresholds. Specifically, the probability of cost-effectiveness for the Lu strategy reached 97.3% at a $150,000 threshold and 12.8% at a $100,000 threshold (Fig. 3). The cost-effectiveness acceptability curve (CEAC) further showed that when drug prices were reduced to 5% and 10% of their original levels, the combination strategy became cost-effective at WTP thresholds of $100,000 and $150,000, respectively (Fig. 4).

### Scenario analysis

In the base-case analysis, lurbinectedin dosing was determined by patient BSA. Due to mismatches between individual dosing requirements and available vial sizes, drug wastage was estimated at 2.18 mg/patient-cycle, corresponding to $4,496.25 /patient-cycle. When drug wastage was excluded, the total cost of the Lu strategy decreased to $332,888.86, yielding savings of approximately $50,000 compared with the base case. The resulting ICER was $606,228.36/QALY, representing a 22% reduction. Under this assumption, an 81% joint price reduction rendered the Lu strategy cost-effective. The CEAC indicated that the Lu strategy became cost-effective at WTP thresholds above $600,000 (Figure S4 in Supplement Materials). In clinical practice, however, complete elimination of drug wastage is unlikely; therefore, measures to reduce rather than eliminate wastage represent more realistic scenarios.

In another scenario, implementing 10% dose rounding improved alignment between prescribed doses and vial sizes. After 10,000 Monte Carlo simulations, the Lu group’s median total cost was $349,245.38, with an associated ICER of $660,350.41/QALY. The Lu strategy would become cost-effective only if the prices of both lurbinectedin and atezolizumab were reduced by 80%. In an alternative scenario assessing VS, wastage rates for lurbinectedin were reduced from 27.2% to 1.2% in high-volume cancer centers and 4% in outpatient chemotherapy clinics settings. This yielded monthly cost savings of $399.72 and $387.00, respectively, with corresponding ICERs of $611,600.47/QALY and $619,593.53/QALY (Table 2).

We also examined the impact of varying the time horizon. When shortened to 5 years, total costs were similar to the base case, but QALYs decreased to 1.07 for the Lu group and 0.90 for the Ate group, resulting in an ICER of $1,336,736.33/QALY. When extended to 15 and 20 years, total costs of the Lu strategy were $390,089.28 and $396,261.81, with corresponding QALYs of 1.40 and 1.52. The resulting ICERs were $554,559.65/QALY and $444,522.09/QALY, respectively, substantially lower than the base case. Results from alternative parametric models used for survival curve fitting and extrapolation are presented in Table S6 in Supplement Materials.

## Discussion

The IMforte clinical trial evaluated the clinical efficacy and safety of lurbinectedin plus atezolizumab; however, the economic implications of this regimen remain unclear. This study assessed the cost-effectiveness of lurbinectedin plus atezolizumab compared with atezolizumab monotherapy as first-line maintenance therapy for ES-SCLC, from the perspective of US payers. Our results showed that although the combination therapy provided additional QALY gains, these benefits were insufficient to offset the substantial increase in costs, rendering the regimen not cost-effective under WTP thresholds in the United States. Even when drug wastage was disregarded, the ICER remained above commonly accepted thresholds. Notably, across all scenarios, QALYs accumulated in the PD state exceeded those in the PF state. Consistently, OWSA identified PD-state utility as one of the most influential parameters. This pattern may be explained by the fact that the combination therapy imposed considerable upfront costs while most patients were still in the PFS state, yet it did not prevent progression. The incremental clinical benefit of lurbinectedin appeared primarily in prolonging survival in the PD state, leading to greater QALY accumulation after progression. Furthermore, with extended time horizons, the benefits of adding lurbinectedin become more apparent in the later stages. In the 5-year scenario analysis, this advantage was not fully realized, as the early accumulation of high costs was not adequately translated into survival benefits, resulting in a markedly higher ICER. In contrast, over a 10- to 20-year horizon, the tail effect of the combination therapy allowed continued QALY accumulation [27, 28], whereas most patients in the monotherapy group had transitioned to the death state, with minimal additional QALYs. As a result, incremental costs plateaued, whereas survival benefits increased, thereby substantially lowering the ICER. Of particular concern, even under a lifetime horizon and after excluding the impact of drug wastage, the ICER of the combination therapy remained above the commonly accepted WTP threshold, suggesting a mismatch between current drug prices and their value. In the base-case scenario, without measures to mitigate wastage, an 85% joint price reduction would be required to achieve cost-effectiveness at a $150,000 threshold. In the most optimistic scenario (ignoring drug wastage), a 65% reduction would suffice. These findings highlight that, in addition to price negotiations, adopting practical strategies to reduce drug wastage is a critical step toward improving the value of the combination therapy.

Drug wastage arises primarily from mismatches between available vial sizes and prescribed doses, and is therefore directly influenced by patient BSA, vial size selection, and drug prices. In this study, the wastage rate of lurbinectedin was approximately 27%, consistent with previously reported ranges of 20–30% for other chemotherapy agents [29]. As a direct consequence, total costs in the Lu group increased by 13%. We explored two mitigation strategies, vial sharing and dose rounding. Results showed that vial sharing offered the greatest potential to reduce total costs and ICERs for the combination regimen, while combining both approaches approximated the ideal scenario of eliminating wastage altogether. Importantly, our vial-sharing assumptions were based on US Pharmacopeia (USP-797) standards, which stipulate that opened vials must be used within six hours [30]. At large cancer centers such as MD Anderson, where daily chemotherapy throughput is more than 3 patients, vial sharing is feasible. However, in community-based chemotherapy clinics or general hospitals, daily patient volume is often insufficient, with an average of fewer than one person per day. For these smaller infusion centers, a “Drug Day” strategy may be necessary, encouraging patients to receive intravenous treatment on the same day to facilitate vial sharing and alleviate the shortage of infusion nurses. Model estimates suggested that setting a Drug Day every three days, ensuring at least two patients receive lurbinectedin infusions simultaneously, could reduce wastage to 3%, yielding an ICER of $619,593.53/QALY. In practice, patient adherence, variability in regional patient volume, or insufficient enrollment between consecutive Drug Days may limit the feasibility of vial sharing. To address these barriers, extending the interval between Drug Days or adopting closed-system transfer devices (CSTDs) could reduce contamination risks and extend vial usability from 6 h to 7 days, allowing more patients to be pooled. However, CSTDs may increase preparation costs by approximately 10% [31]. Additionally, dynamically scheduling Drug Days based on anticipated patient numbers could enhance efficiency. From a payment perspective, alternative reimbursement models such as patient-specific dosing charges or outcomes-based delayed payment may facilitate risk-sharing among manufacturers, payers, and patients.

To our knowledge, no prior study has evaluated the cost-effectiveness of combining lurbinectedin with immunotherapy versus immunotherapy alone in ES-SCLC. Zhou et al. assessed the cost-effectiveness of atezolizumab plus EP compared with chemotherapy alone as first-line treatment for ES-SCLC [32]. Their findings indicated that the combination was not cost-effective, and achieving cost-effectiveness would require a 95% reduction in the price of atezolizumab, which is consistent with the conclusions of our study. In addition, drug wastage has been widely discussed since the early 2000s; however, previous studies have not examined the impact of drug wastage and waste-reduction strategies on the cost-effectiveness of ES-SCLC treatments. This issue imposes a substantial financial burden on the US healthcare system, while evidence shows that interventions aimed at reducing wastage can be highly effective [33], suggesting considerable potential for improving efficiency and value.

## Limitation

This study has several limitations. First, due to the absence of ES-SCLC specific EQ-5D-5 L utility values, we adopted estimates from US patients with NSCLC as model inputs. Although this approach is consistent with prior studies, the interchangeability of NSCLC and SCLC utility values has not been validated and should be regarded as a key methodological assumption. Sensitivity analyses, however, suggested that this assumption did not affect the robustness of the results. Second, estimates of drug wastage and waste-reduction strategies were based on theoretical projections and have not yet been externally validated using real-world data. Third, due to limited information, dose reductions caused by treatment-related toxicities were not incorporated. Fourth, cross-patient vial sharing is not recommended in certain countries, such as India, which may limit the generalizability of our findings [34]. Finally, dose rounding of ± 10% for cytotoxic drugs is widely recommended in clinical practice [33], and several studies have demonstrated minimal impact on safety and efficacy [35, 36]. Nevertheless, the tolerance of lurbinectedin to dose rounding and its potential impact on efficacy remain uncertain and require confirmation through real-world evidence.

## Conclusion

The introduction of lurbinectedin has expanded first-line maintenance therapy options for ES-SCLC patients and demonstrated strong synergy with immunotherapy, significantly improving survival outcomes. However, the high cost of lurbinectedin imposes a substantial economic burden, rendering the combination regimen not cost-effective from the perspective of the US healthcare system. Furthermore, the considerable issue of drug wastage exacerbates concerns about affordability. Effective policy interventions to mitigate drug wastage, along with value-based and volume-based pricing strategies, should be prioritized to enhance the economic viability of this regimen.

## Supplementary Information

Below is the link to the electronic supplementary material.

Supplementary Material 1

## Data Availability

The data that support the findings of this study are available from the corresponding author upon reasonable request.

## Abbreviations

AE: Adverse events
AIC: Akaike Information Criterion
Ate: Atezolizumab
BSA: Body surface area
BSC: Best supportive care
CEAC: Cost-effectiveness acceptability curve
CMS: Centers for Medicare & Medicaid Services
CT: Computed tomography
EP: Etoposide plus platinum
ES-SCLC: Extensive-stage small-cell lung cancer
ICER: Incremental cost-effectiveness ratio
IPD: Individual patient data
KM: Kaplan-Meier
Lu: Lurbinectedin
NCCN: National Comprehensive Cancer Network
NSCLC: Non-small cell lung cancer
OS: Overall survival
PD: Progressive disease
PF: Progression-free
PFS: Progression-free survival
PSA: Probabilistic sensitivity analysis
QALY: Quality-adjusted life year
SCLC: Small-cell lung cancer
STING: Stimulator of interferon genes
VS: Vial sharing
WTP: Willingness-to-pay
