# Supplementary material for: Cost-effectiveness of lurbinectedin with atezolizumab for extensive-stage small-cell lung cancer in the United States: impact of drug wastage reduction on economic outcomes
Source: BMC Health Serv Res. 2026 May 4;26:839. doi: 10.1186/s12913-026-14653-5 (PMC13274110; doi:10.1186/s12913-026-14653-5)
Supplement: Supplementary file 1 — Supplementary Material 1 [file 12913_2026_14653_MOESM1_ESM.docx]

***Supplementary Materials***

Table S2 Summary of medication ratios, dosages and wastage.

| Drugs | Proportion receiving relevant treatment in Lu group | Proportion receiving relevant treatment in Ate group | Usage | Minimum bottle size (mg) | Waste rate | Wastage outcome of US dollar ($/patient-cycle) | Wastage outcome of milligrams (mg/patient-cycle) |
| --- | --- | --- | --- | --- | --- | --- | --- |
| Lurbinectedin | - | - | 3.2 mg/m^2^, iv, 1/21d, Q3W | 4 | 27.2% | 4488.00 | 2.18 |
| Atezolizumab ^a^ | - | - | 1200 mg, iv, 1/21d, Q3W | 1200 | 0.0% | - | - |
| Carboplatin | 0.16 | 0.11 | AUC 5mg/mL/min, iv, 1/21d, Q3W | 50 | 5.0% | 3.14 | 25.00 |
| Etoposide | 0.14 | 0.1 | 100mg/m^2^, iv, d1-3/21d, Q3W | 100 | 9.0% | 5.56 | 54.00 |
| Topotecan | 0.1 | 0.16 | 1.5 mg/m^2^, iv 30 min, d1-5/21d, Q3W | 4 | 9.0% | 222.25 | 6.35 |
| Irinotecan | 0.1 | 0.14 | 350mg/m^2^, iv, 1/21d, Q3W | 100 | 9.0% | 19.10 | 63.00 |
| Atezolizumab ^b^ | 0.08 | 0.04 | 1200 mg, iv, 1/21d, Q3W | 1200 | 0.0% | - | - |

Abbreviations: AUC: area under curve; iv: intravenous; Q3W: every 3 weeks; d: day.

a: atezolizumab as first-line treatment

b: atezolizumab as a subsequent anti-tumor treatment

Table S3 Summary of follow-up methods.

| Time horizon | Examination items | Follow-up frequency |
| --- | --- | --- |
| First year | Functional MRI,  Pet image w/ct full body,  Blood test,  Breathing capacity test | once every 3 cycles |
| Second to third year |  | once every 6 cycles |
| Fourth to fifth year |  | once every 9 cycles |
| After the sixth year |  | once every 18 cycles |

Table S4 : Incidence of grade 3-4 adverse events included in the model.

| Types of adverse events (grade 3-4, %) | Lurbinectedin | Atezolizumab |
| --- | --- | --- |
| Anaemia | 8 | 1 |
| Decreased neutrophil count | 7 | 0 |
| Decreased platelet count | 7 | 0 |
| Neutropenia | 5 | 0.4 |
| Thrombocytopenia | 5 | 0.4 |

Table S5 Goodness-of-fitness results.

|  | OS of lurbinectedin | | | OS of atezolizumab | | | PFS of lurbinectedin | | | PFS of atezolizumab | | |
| --- | --- | --- | --- | --- | --- | --- | --- | --- | --- | --- | --- | --- |
| Distribution | LnL | Params | AIC | LnL | Params | AIC | LnL | Params | AIC | LnL | Params | AIC |
| Exponential | -64.9326 | 1 | 131.8652 | -73.74224 | 1 | 149.4845 | -73.11102 | 1 | 148.222 | -100.2436 | 1 | 202.4872 |
| Weibull | -55.32906 | 2 | 114.6581 | -63.94705 | 2 | 131.8941 | -69.5377 | 2 | 143.0754 | -100.2636 | 2 | 204.5273 |
| Gamma | -55.79695 | 2 | 115.5939 | -62.99519 | 2 | 129.9904 | -67.6196 | 2 | 139.2392 | -100.0782 | 2 | 204.1564 |
| Log-Normal | -62.40493 | 2 | 128.8099 | -64.8047 | 2 | 133.6094 | -60.57218 | 2 | 125.1444 | -71.32409 | 2 | 146.6482 |
| Gompertz | -57.10274 | 2 | 118.2055 | -68.56009 | 2 | 141.1202 | -72.70253 | 2 | 149.4051 | -91.83485 | 2 | 187.6697 |
| Log-Logistic | -56.64063 | 2 | 117.2813 | **-62.47519** | **2** | **128.9504** | -63.37605 | 2 | 130.7521 | -72.35862 | 2 | 148.7172 |
| Generalized Gamma | -55.30379 | 3 | 116.6076 | -62.69351 | 3 | 131.387 | -60.37074 | 3 | 126.7415 | -55.86631 | 3 | 117.7326 |
| FP1-1 | -62.3943 | 2 | 128.7886 | -73.12037 | 2 | 150.2407 | -74.19513 | 2 | 152.3903 | -99.95616 | 2 | 203.9123 |
| FP1-2 | -56.89317 | 2 | 117.7863 | -62.96378 | 2 | 129.9276 | -66.68311 | 2 | 137.3662 | -106.3866 | 2 | 216.7732 |
| FP2-1 | -53.9573 | 3 | 113.9146 | -64.75239 | 3 | 135.5048 | -73.47652 | 3 | 152.953 | -98.41306 | 3 | 202.8261 |
| FP2-2 | -54.01484 | 3 | 114.0297 | -63.95382 | 3 | 133.9076 | -72.43917 | 3 | 150.8783 | -96.43435 | 3 | 198.8687 |
| RCS1 | **-54.15056** | **3** | **114.3011** | -63.99856 | 3 | 133.9971 | -71.88557 | 3 | 149.7711 | -94.59211 | 3 | 195.1842 |
| RCS2 | -54.13809 | 4 | 116.2762 | -63.58003 | 4 | 135.1601 | -65.62191 | 4 | 139.2438 | -55.78045 | 4 | 119.5609 |
| RP-hazard-1 | -55.329 | 2 | 114.658 | -62.74878 | 3 | 131.4976 | **-59.06833** | **5** | **128.1367** | -55.33787 | 3 | 116.6757 |
| RP-hazard-2 | -55.14685 | 3 | 116.2937 | -63.94708 | 2 | 131.8942 | -61.61085 | 6 | 135.2217 | -57.51266 | 4 | 123.0253 |
| RP-odds-1 | -54.57954 | 3 | 115.1591 | -62.47532 | 2 | 128.9506 | -59.94056 | 5 | 129.8811 | -54.91691 | 3 | 115.8338 |
| RP-odds-2 | -56.64109 | 2 | 117.2822 | -62.44085 | 3 | 130.8817 | -61.81198 | 6 | 135.624 | -57.1243 | 4 | 122.2486 |
| RP-normal-1 | -54.82059 | 3 | 115.6412 | -62.58929 | 3 | 131.1786 | -60.57219 | 2 | 125.1444 | **-54.33705** | **3** | **114.6741** |
| RP-normal-2 | -54.81796 | 4 | 117.6359 | -64.80475 | 2 | 133.6095 | -60.38609 | 3 | 126.7722 | -54.27943 | 4 | 116.5589 |

Abbreviations: OS: Overall survival; PFS: Progression-free survival; LnL: Log-likelihood; Params: Parameters; AIC: Akaike information criterion; FP: Fractional polynomial; RCS: Restricted cubic spline models; RP: Royston-Parmar models. Bold data means that this model is the model we finally selected.

Table S6 Summary results of scenario analysis.

| Incremental Cost ($) | Incremental QALYs | ICER ($/QALY) |
| --- | --- | --- |
| Base: RCS1 for OS; RP-hazard-1 for PFS | | |
| 229,342.16 | 0.30 | 773,428.44 |
| Scenario (a): Log-logistic for OS; RP-hazard-1 for PFS | | |
| 224,978.19 | 0.21 | 1,061,316.86 |
| Scenario (b): RCS2 for OS; RP-hazard-1 for PFS | | |
| 237,269.44 | 0.45 | 527,445.14 |
| Scenario (c): RP-odds-2 for OS; RP-hazard-1 for PFS | | |
| 224,967.98 | 0.21 | 1,062,241.92 |
| Scenario (d): RCS1 for OS; Gen-gamma for PFS | | |
| 245,470.25 | 0.30 | 822,898.30 |
| Scenario (e): RCS1 for OS; RP-odds-1 for PFS | | |
| 271,571.36 | 0.30 | 901,724.73 |
| Scenario (f): RCS1 for OS; RP-normal-2 for PFS | | |
| 243,541.51 | 0.30 | 817,013.24 |

Abbreviations: OS: Overall survival; PFS: Progression-free survival; QALY: Quality-adjusted life year

(a)


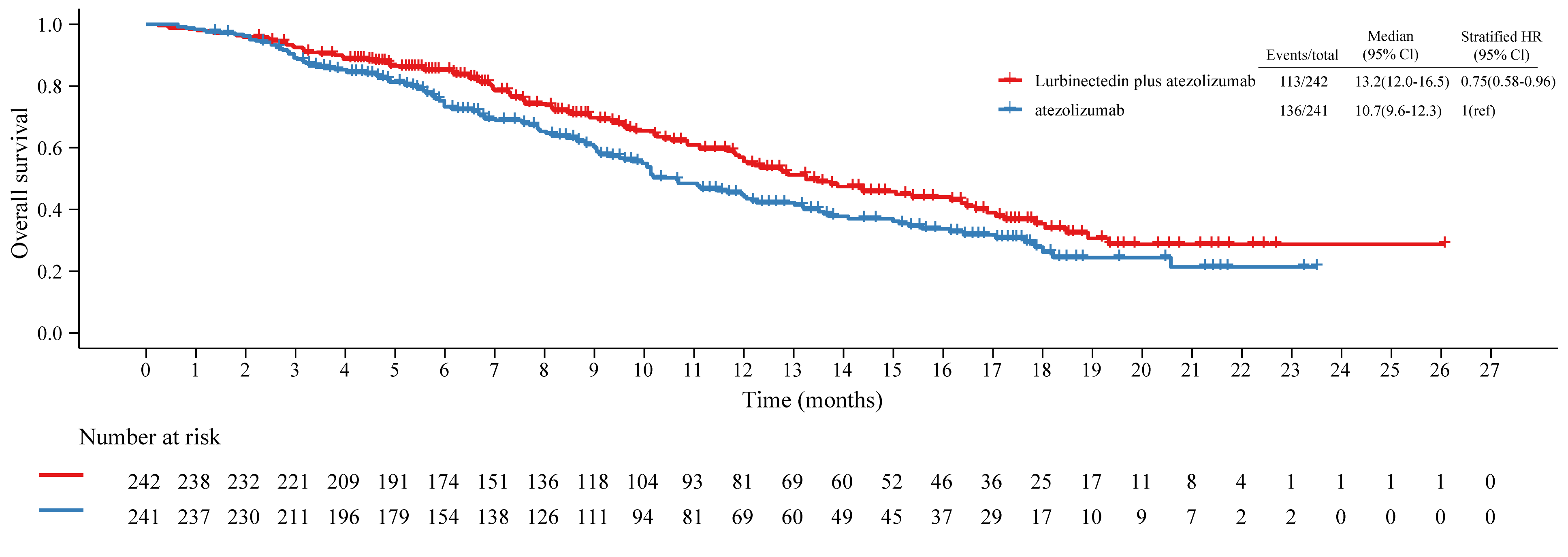
 (b)


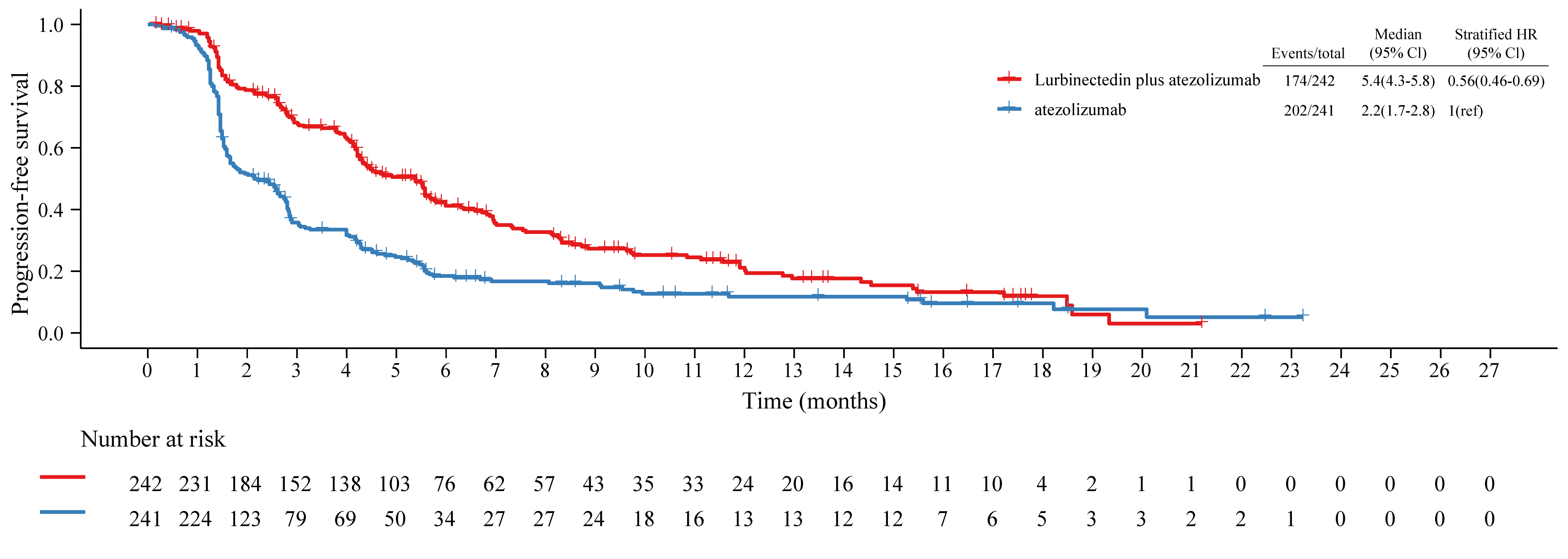
 Figure S1 Reconstruction of Kaplan Meier survival curve. OS curve (a). PFS curve (b).

OS, overall survival; PFS, progression-free survival.

(a)


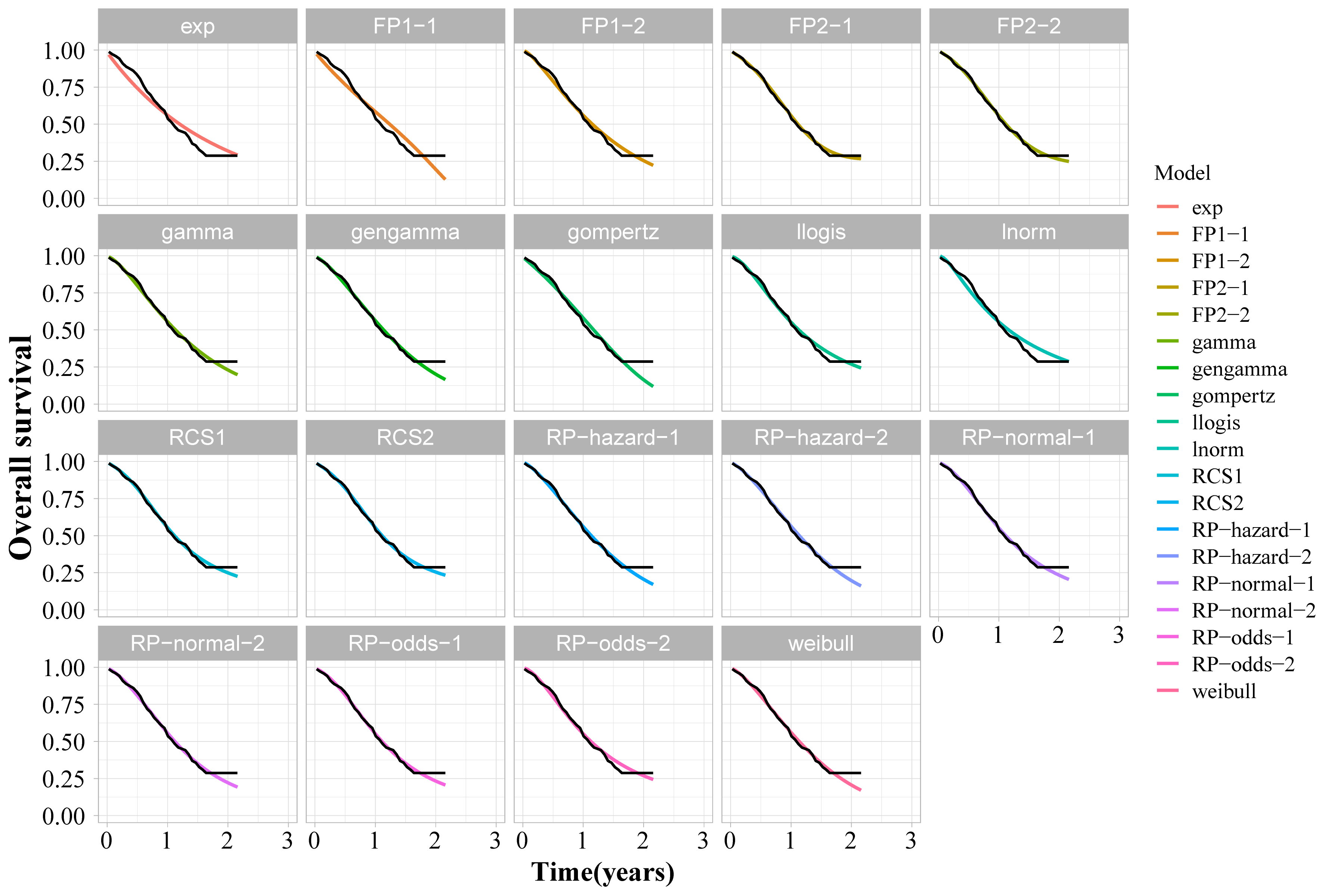


(b)


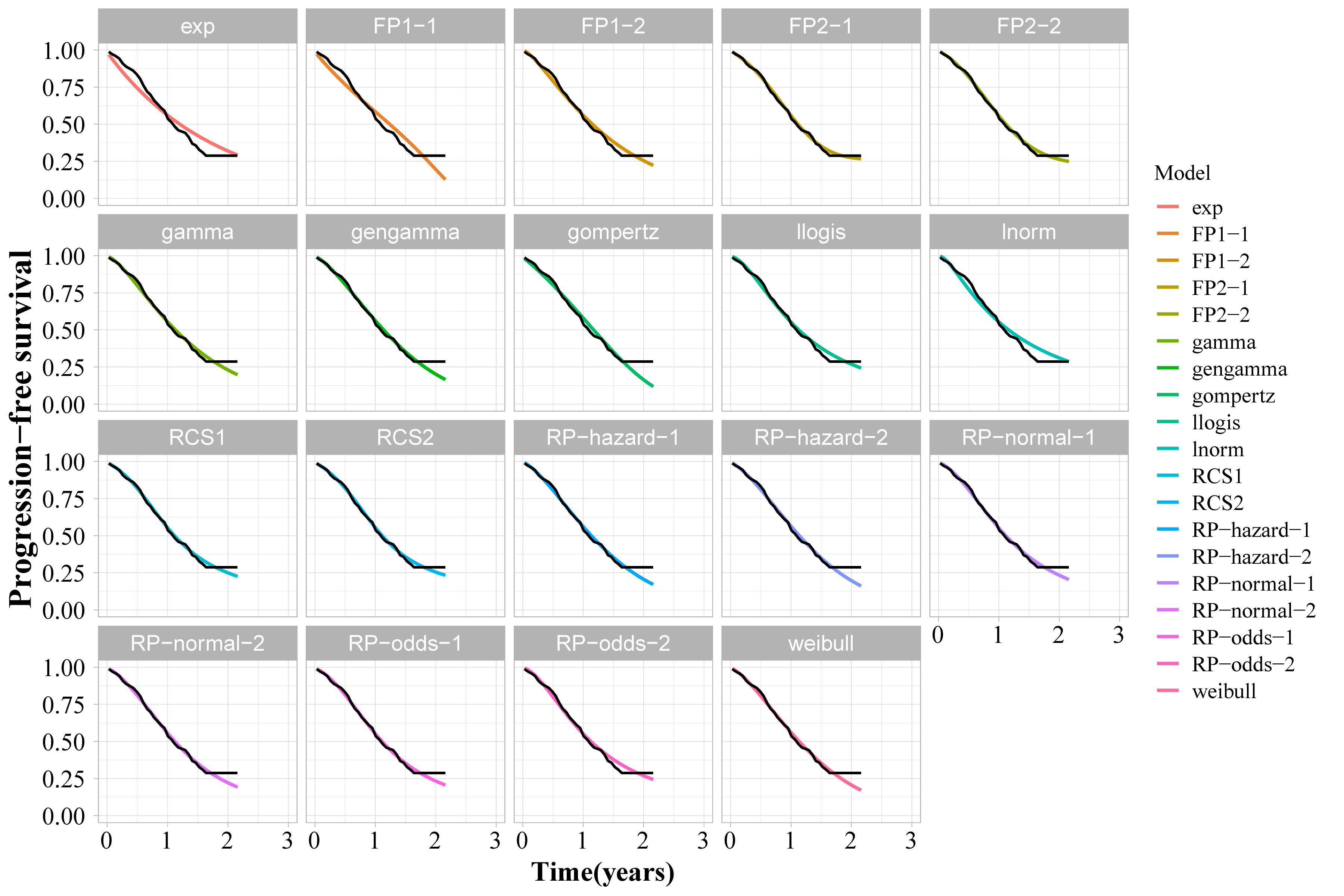


(c)


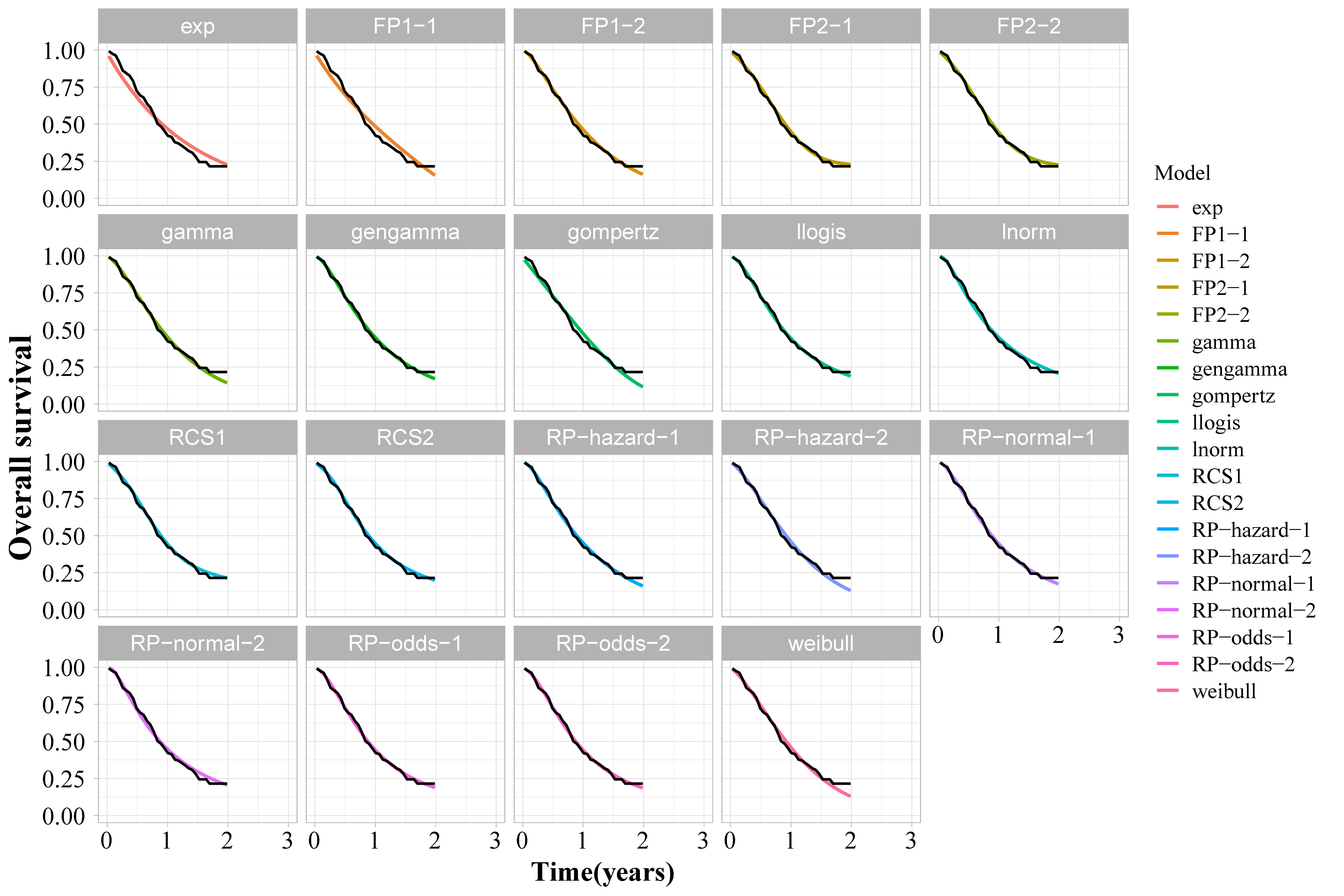


(d)


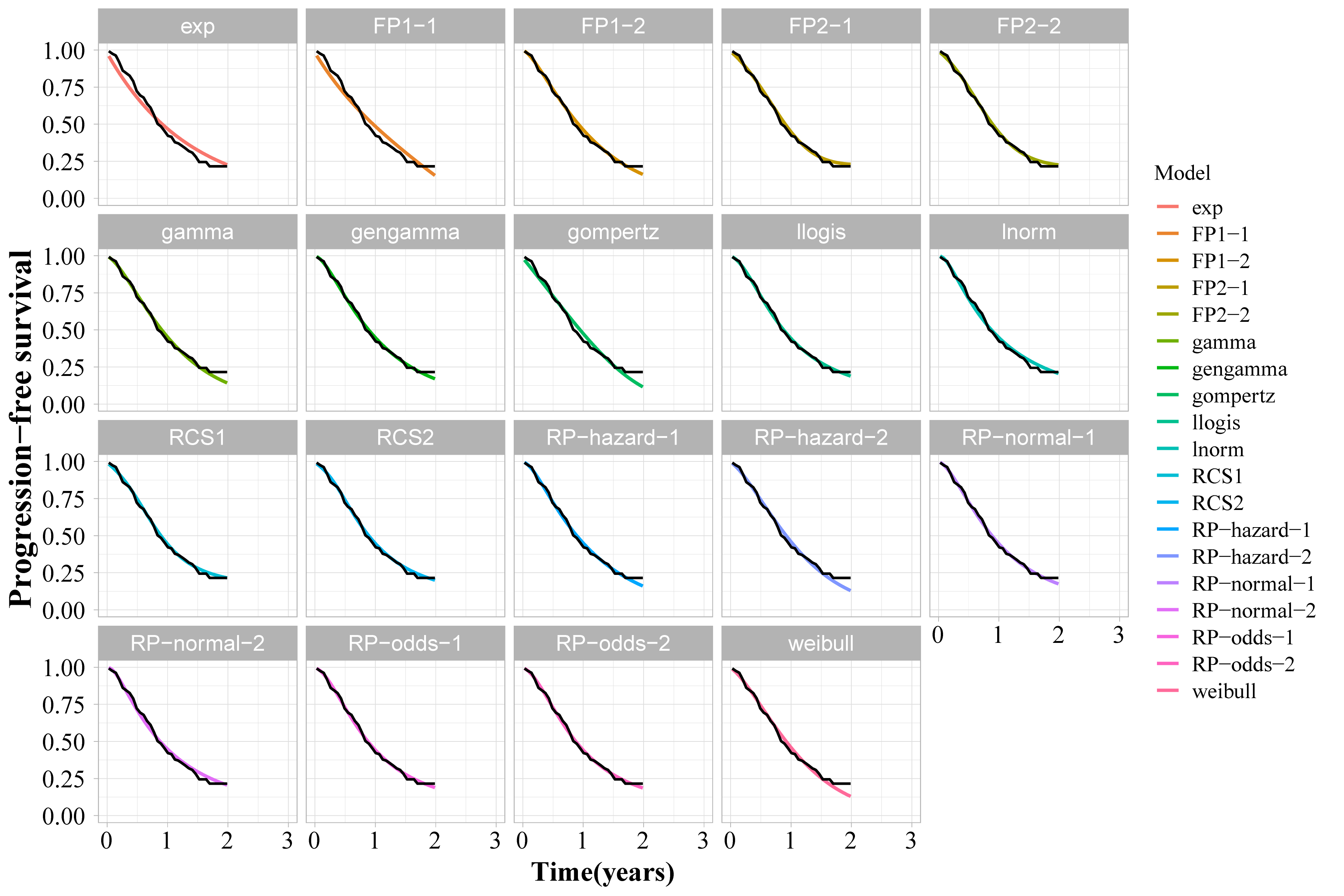


Figure S2 Survival plots showing the goodness-of-fit. OS of lurbinectedin. (a), OS of atezolizumab (b), PFS of lurbinectedin (c), PFS of atezolizumab (d) Abbreviations: OS: overall survival; PFS: progression-free survival; Black line shows the original KM curves


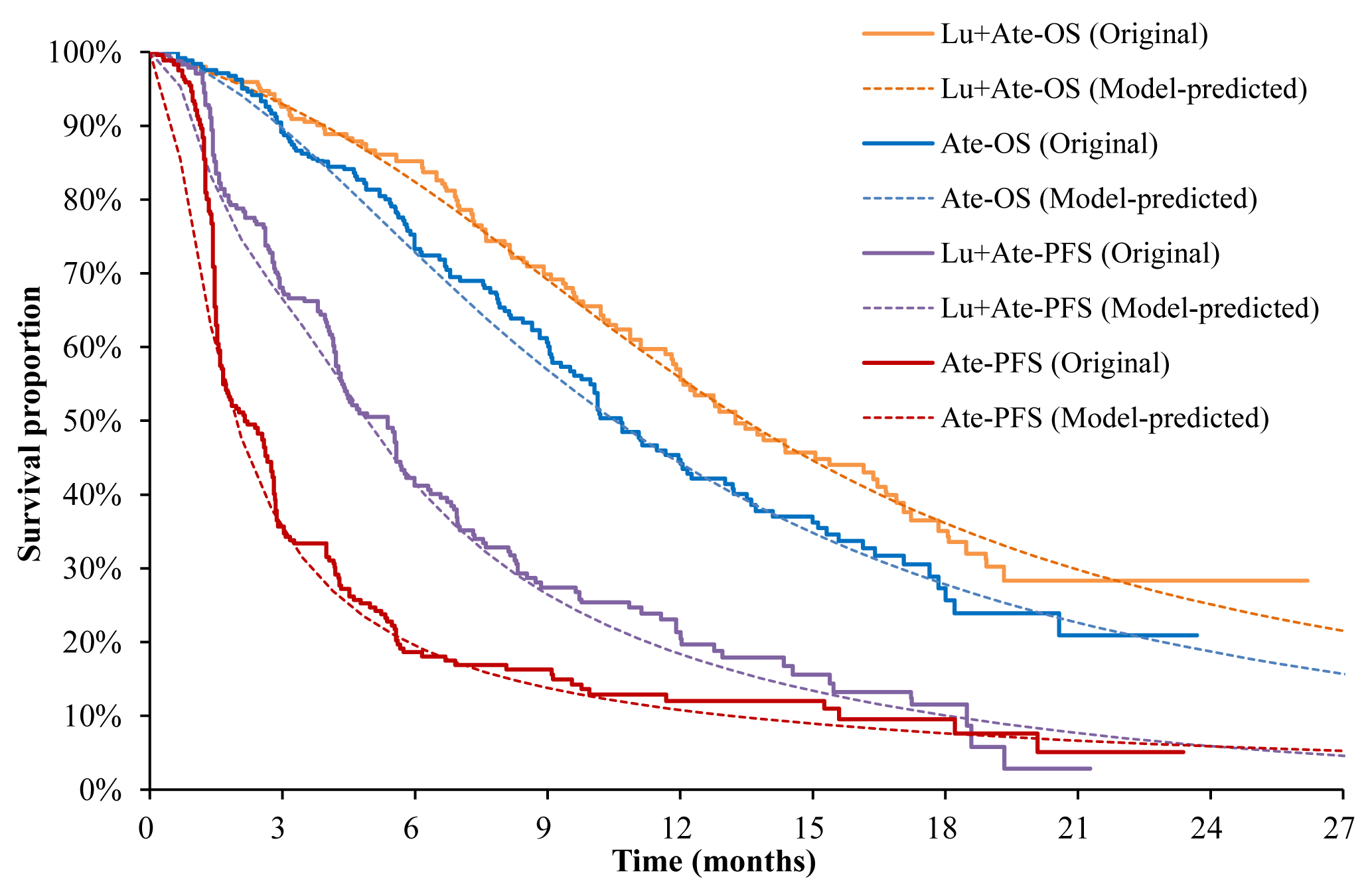


Figure S3 Kaplan-Meier Curves for OS and PFS with Model-Predicted Fits in Lu+Ate and Ate Groups.

OS, overall survival; PFS, progression-free survival; Lu+Ate, lutetium plus atezolizumab; Ate, atezolizumab

(a)


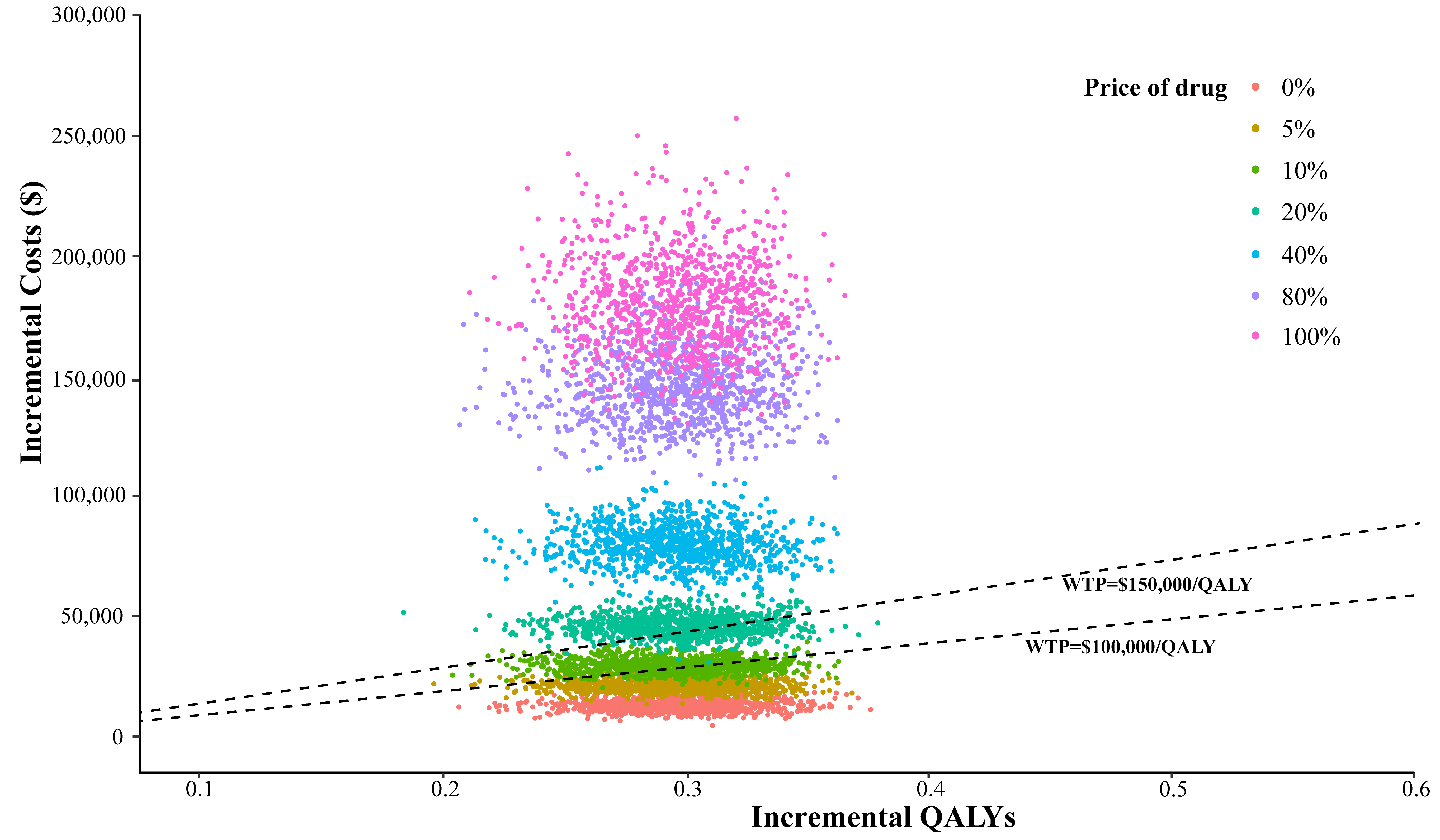


(b)


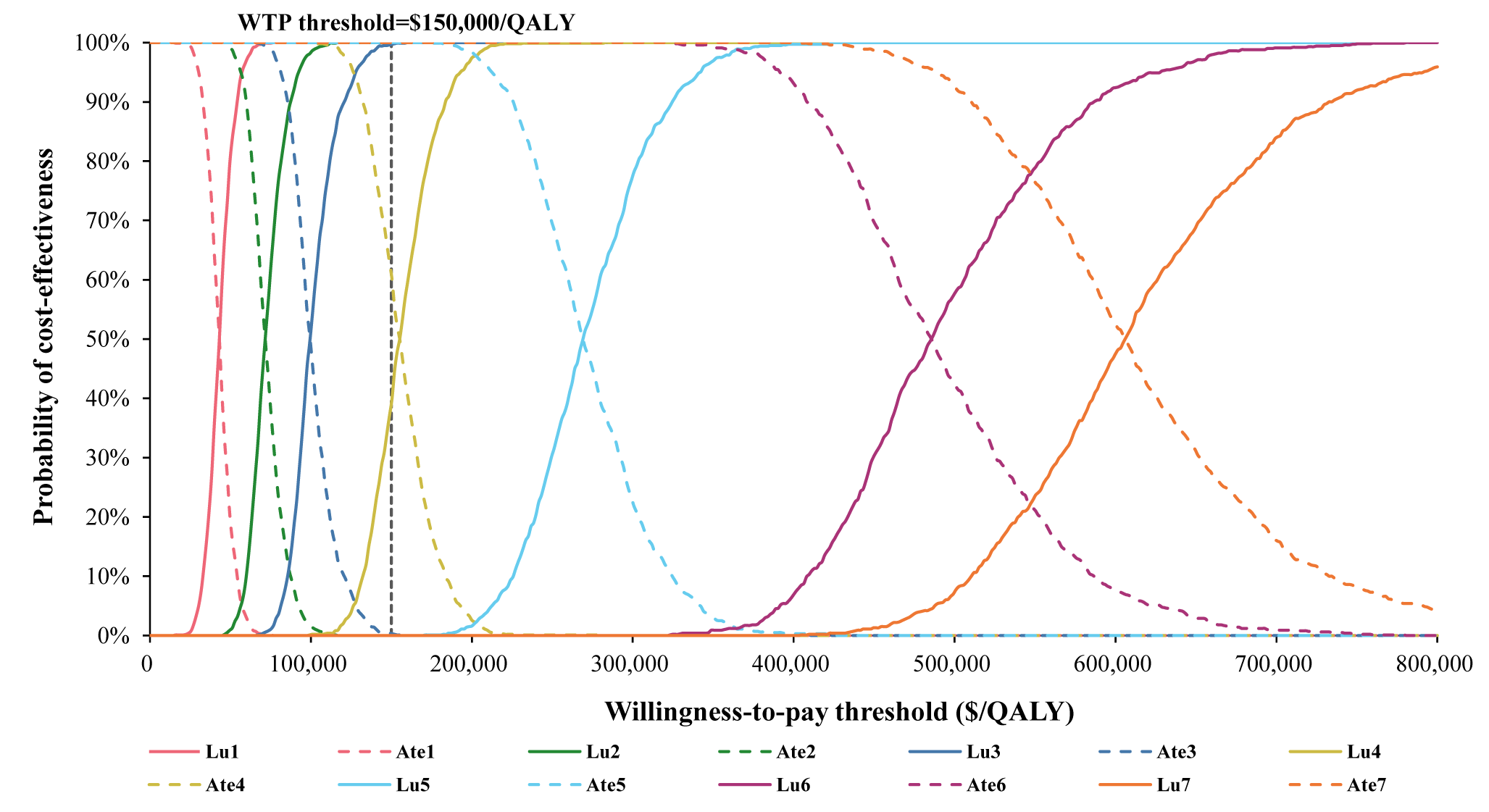


Figure S4 Probabilistic sensitivity analysis for ignoring drug wastage. Scatter plots for probabilistic sensitivity analysis (a), Cost-effectiveness acceptability curves (b).

Abbreviations: WTP: willingness-to-pay; QALY: Quality-adjusted life year; Lu: lurbinectedin; Ate: atezolizumab; 0-7 refers to the price of lurbinectedin and atezolizumab change from 0%, 5%, 10%, 20%, 40%, 80% to 100%.
